# Acute care bundles should be used for patients with intracerebral haemorrhage: An expert consensus statement

**DOI:** 10.1177/23969873231220235

**Published:** 2023-12-27

**Authors:** Adrian R Parry-Jones, Susann J Järhult, Natalie Kreitzer, Andrea Morotti, Danilo Toni, David Seiffge, Alexander David Mendelow, Hiren Patel, Hens Bart Brouwers, Catharina JM Klijn, Thorsten Steiner, Walter Brian Gibler, Joshua N Goldstein

**Affiliations:** 1 Geoffrey Jefferson Brain Research Centre, Manchester Academic Health Science Centre, Northern Care Alliance & University of Manchester, Manchester, UK; 2 Department of Medical Sciences, Uppsala University, Emergency Department, Uppsala University Hospital, Uppsala, Sweden; 3 Department of Emergency Medicine, University of Cincinnati College of Medicine, Cincinnati, OH, USA; 4 Neurology Unit, Department of Clinical and Experimental Sciences, University of Brescia, Brescia, Italy; 5 Emergency Department Stroke Unit, Policlinico Umberto I, University La Sapienza Rome, Italy; 6 Department of Neurology, Inselspital, University Hospital and University of Bern, Bern, Switzerland; 7 Neurosurgery Trials Group, Newcastle University, Newcastle, UK; 8 Department of Neurosurgery, Elisabeth-TweeSteden Hospital, Tilburg, The Netherlands; 9 Department of Neurology, Donders Institute for Brain, Cognition and Behaviour, Radboud University Medical Centre, Nijmegen, The Netherlands; 10 Departments of Neurology, Klinikum Frankfurt Höchst, Frankfurt and Heidelberg University Hospital, Heidelberg, Germany; 11 Department of Emergency Medicine, Massachusetts General Hospital, Boston, MA, USA

**Keywords:** Intracerebral haemorrhage, oral anticoagulants, blood pressure, haematoma evacuation, hyperglycaemia, fever, care bundles

## Abstract

**Purpose:**

Intracerebral haemorrhage (ICH) is the most devastating form of stroke and a major cause of disability. Clinical trials of individual therapies have failed to definitively establish a specific beneficial treatment. However, clinical trials of introducing care bundles, with multiple therapies provided in parallel, appear to clearly reduce morbidity and mortality. Currently, not enough patients receive these interventions in the acute phase.

**Methods:**

We convened an expert group to discuss best practices in ICH and to develop recommendations for bundled care that can be delivered in all settings that treat acute ICH, with a focus on European healthcare systems.

**Findings:**

In this consensus paper, we argue for widespread implementation of formalised care bundles in ICH, including specific metrics for time to treatment and criteria for the consideration of neurosurgical therapy.

**Discussion:**

There is an extraordinary opportunity to improve clinical care and clinical outcomes in this devastating disease. Substantial evidence already exists for a range of therapies that can and should be implemented now.

## Graphical abstract

**Graphical abstract:**
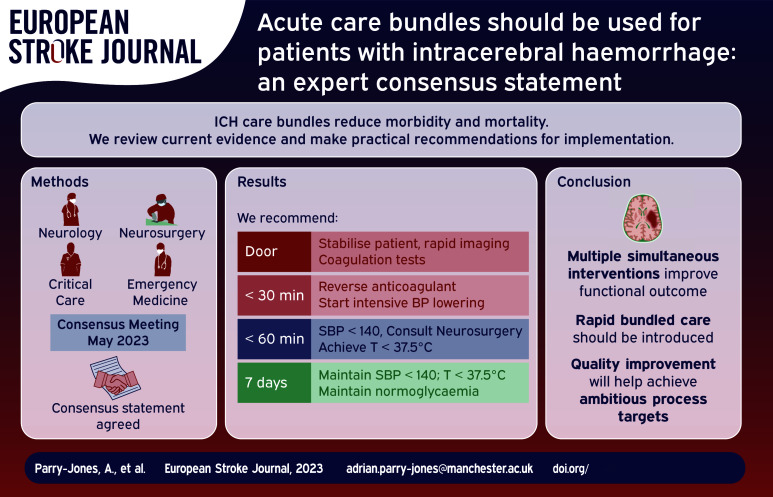

## Introduction

Intracerebral haemorrhage (ICH) represents a major global health burden.^1^ Traditionally, the lack of definitive clinical trial data for specific ICH treatments has led to pessimism.^2^ However, recent trials have given cause for optimism for the future of ICH care. The third Intensive Care Bundle with Blood Pressure Reduction in Acute Cerebral Haemorrhage Trial (INTERACT3) demonstrated that implementation of a goal-directed care bundle reduces the odds of a poor functional outcome.^3^ A similar care bundle approach has been associated with a significant reduction in mortality in a UK hospital.^4^ These studies suggest that all hospitals and regional acute care systems should now incorporate a care bundle approach when managing patients with ICH. Here, we consider how a care bundle might be implemented.

## Methods

An expert panel with representation from Emergency Medicine (JG, NK, WBG, SJ), Stroke Neurology (APJ, TS, DS, AM, DT, CK), Neurocritical Care (JG, NK, TS), and Neurosurgery (HP, HBB, DM) was convened by the Emergency Medicine Cardiac Research and Education Group (EMCREG)-International and met for a panel discussion in May 2023. Consensus was reached on key components of an ICH care bundle, a writing group was convened, and all members of the panel contributed to the current manuscript.

## Initial evaluation of patients with potential ICH

For patients with acute stroke symptoms, the initial evaluation should be rapid and focused.^5^ This should include establishing onset time, use of any antithrombotic medications, a focused physical exam including ABCDE algorithm (airway, breathing, circulation, disability, exposure), and rapid neuroimaging. Non-invasive angiography should be considered, using tools such as the DIAGRAM score to select patients with a higher probability of finding a macrovascular cause.^5,6^ In addition to routine laboratory testing, specialised coagulation tests including thrombin time and anti-factor-Xa levels, if rapidly available, should be considered in patients taking direct oral anticoagulants (DOACs). Point-of-care tests are well-established for the international normalised ratio (INR) and although urine tests for the presence of DOACs are available, they provide only a binary (positive or negative) result and may be positive at very low DOAC concentrations.^7^

## Components of a care bundle for ICH

Once the patient has been stabilised and diagnosis confirmed, ICH-specific care should be rapidly initiated (Table 1).

**Table 1.** Individual interventions for possible inclusion in an acute, ICH-specific bundle of care.

Intervention	Criteria for treatment	Recommended process targets	Supporting guidelines/key evidence
Anticoagulant reversal	PCC and vitamin K (VKA antagonist): INR ⩾ 1.3 Andexanet alfa: currently taking apixaban or rivaroxaban and last dose taken ⩽18 h Idarucizumab: currently taking dabigatran PCC (DOACs): taking a DOAC and specific reversal agent unavailable or unlicenced for specific agent	Door-to-needle time ⩽ 30 min	1. ESO anticoagulant- associated ICH guideline (2019)^11^ 2. AHA/ASA ICH guideline (2022)^5^ 3. REVERSE-AD^14^ 4. ANNEXA-4^15^
Intensive blood pressure reduction	⩽6 h after symptom onset: SBP ⩾ 150 mmHg ⩾6 h after symptom onset or unknown onset: uncertain, consider if SBP ⩾ 150 mmHg	Treatment target ⩽ 140 mmHg, maintained for 7 days Avoid large (>90 mmHg) initial drops on SBP Door-to-first antihypertensive: ⩽30 min Door-to-target: ⩽60 min	1. ESO BP guideline (2021)^21^ 2. AHA/ASA ICH guideline (2022)^5^ 3. INTERACT2^47^ & 3^3^
Surgical evacuation of haematoma and/or external ventricular drainage	Decision to operate on a case-by-case basis by a multi-disciplinary team. Local criteria should be established to identify patients where a consultation with neurosurgery must occur, for example: Patients with a pre-morbid mRS of ⩽2, reasonable prognosis and one or more of: 1. GCS ⩽ 13 2. Supratentorial ICH volume ⩾ 20 mL 3. Posterior fossa ICH 4. Obstruction of third and fourth ventricle(s)	100% of patients meeting consultation criteria are discussed with neurosurgery ⩽50% of patients not meeting consultation criteria are discussed with neurosurgery within 60 min of arrival.	1. ESO ICH guideline (2014)^33^ 2. AHA/ASA ICH guideline (2022)^5^
Control of glucose	Non-diabetic patients: Blood glucose > 7.8 mmol/L in first 7 days Diabetic patients: Blood glucose > 10 mmol/L in first 7 days	Non-diabetic patients: maintain blood glucose between 6.1 and 7.8 mmol/L for ⩾90% of measurements in first 7 days Diabetic patients: maintain blood glucose between 7.8 and 10 mmol/L for ⩾90% of measurements in first 7 days For both groups, optimise protocols to avoid hypoglycaemia	1. INTERACT3^3^ 2. QASC^37^ AHA/ASA guideline (2022)^5^
Control of temperature	Monitor body temperature every 4 h for 7 days and initiate anti-pyretic treatment if temperature ⩾ 37.5°C	Achieve normothermia (<37.5°C) within 1 h of starting treatment	1. INTERACT3^3^ 2. QASC^37^ 3. AHA/ASA guideline (2022)^5^

AHA/ASA: American Heart Association/American Stroke Association; DOAC: direct oral anticoagulant; ESO: European Stroke Organisation; GCS: Glasgow Coma Scale; ICH: intracerebral haemorrhage; INR: international normalised ratio; PCC: prothrombin complex concentrate; SBP: systolic blood pressure; VKA: vitamin-K antagonist; mRS: modified Rankin Scale.

### Anticoagulation reversal

Haematoma expansion (HE) is associated with worse outcomes after ICH and occurs in around 30% of all ICH patients within 3 h of onset.^8^ The likelihood of HE increases to 54%in anticoagulated patients^9^; thus, a critical component of any ICH care bundle is early anticoagulation reversal.

*Vitamin K antagonists (VKAs)*: Rapid VKA reversal requires Vitamin K administration in addition to coagulation factor repletion. Prothrombin complex concentrates (PCCs) reduce the INR quickly and efficiently,^10^ and are recommended by the European Stroke Organization and the American Heart Association.^5,11^ The benefit of this agent may be time dependent; in a large multicentre retrospective study of VKA-ICH, those receiving PCCs within 4 h (and had a systolic blood pressure [SBP] < 160 mmHg) had a rate of HE of 18%, compared to 44% in patients not achieving these values.^12^

*DOACs* have become the first line anticoagulant for most patients,^13^ transforming the approach to anticoagulation reversal in ICH.

- Factor IIa inhibitors (dabigatran): For this agent, there is a highly specific reversal agent, idarucizumab, a monoclonal antibody fragment that binds dabigatran and appears to be effective for haemostasis.^14^
- Factor Xa inhibitors (rivaroxaban, apixaban, edoxaban): The specific reversal agent available is andexanet alfa, a recombinant modified version of human Factor X. Of note, the use of andexanet alfa to reverse edoxaban is currently off-label in many countries. Andexanet alfa binds Factor Xa inhibitors and appears to be effective for haemostasis in patients within 18 h of their last DOAC dose.^15^ The ANNEXa-I randomised controlled trial (NCT03661528) has compared andexanet alfa with standard care (mainly PCC) in ICH patients within 6 h of symptom onset. Although not yet published, it has been presented at a major international conference and superior haemostatic efficacy with andexanet alfa is reported, at the cost of an increase in thrombotic complications, especially in those with a prior history of stroke or myocardial infarction.^16^

Time is critical to anticoagulation reversal. Similar to thrombolysis in ischaemic stroke,^17^ door-to-needle (DTN) time metrics should be targeted with ICH, because the risk of HE is highest in the hours following ICH and providing early reversal maximises the benefits. In contrast, the costs and adverse event risks may be the same irrespective of time; therefore, earlier treatment may maximise the risk-benefit ratio. We recommend hospitals implement an aspirational DTN target of under 30 min. This is much shorter than current common practice and a more relaxed target may be needed initially, depending on current performance. However, aspiring to achieve a challenging DTN goal for all cases will likely instil a sense of urgency and ensure that reversal is achieved as quickly as possible. Key steps in reducing DTN time include expediting imaging and IV placement, developing streamlined protocols with key stakeholders, and training staff in drug reconstitution. These components have all been effective steps in reducing DTN time below 30 min in ischaemic stroke.^18^

Unlike INR for patients taking VKAs, DOACs do not have a laboratory test that is widely and rapidly available. Therefore, clinicians rely on history, which may be challenging to obtain in ICH. Important information needed for decision-making in patients taking a DOAC includes dose, time of last intake, and kidney function. To avoid delay, we recommend empirical reversal, unless there is a concern about compliance, dosage, timing of last dose, and risks of thrombotic complications.

### Blood pressure reduction

Elevated SBP is common in ICH and associated with an increased risk of HE and poor outcome.^19^ Control of hypertension in the acute phase reduces the risk of HE and since the majority of HE occurs early, the beneficial effects of intensive SBP reduction are likely to be time-dependent.^8^ The recent INTERACT3 trial included a goal SBP < 140 mmHg within the first hour and the number needed to treat (NNT) was 35 (95% CI 15 to infinity) for the care bundle to prevent one patient from death or major disability.^3^ It is important to note that many patients did not reach this goal and achieved an average SBP of 150 mmHg, highlighting the value of early SBP reduction even if target SBP is not achieved.

In order to ensure maximum benefit from BP reduction, antihypertensive medications should be initiated within 30 min and target SBP achieved within 60 min.^5,20,21^ The optimal SBP target in patients with acute ICH remains a matter of debate. Current best evidence suggests:

- Patients within 6 h of onset and SBP of 150–220 mmHg: Aiming for SBP < 140 mmHg appears to improve outcome, even if the average achieved SBP is 150 mmHg.
- For >6 h from onset evidence is limited, but AHA guidelines recommend a SBP target of 130–150 mmHg^5^ and ESO guidelines a SBP target of 110–140 mmHg.^21^

Target SBP should be achieved smoothly, avoiding SBP fluctuations and large drops (>90 mmHg), especially in the first hour.^21^ There is limited evidence to guide SBP treatment in patients with severely impaired consciousness, high volume (e.g. >60 mL) haemorrhages and admission SBP > 220 mmHg.

Once target SBP has been achieved, it should be maintained for the next 7 days.^3^ Good BP control is a cornerstone of minimising risk of recurrent ICH, as well as other cardiovascular events.^22^

### Neurosurgical management

ICH volume is a powerful predictor of functional outcome, and therefore surgical evacuation may improve outcome by reducing mass effect, lowering intracranial pressure, and lessening secondary injury. Many surgical trials have shown trends towards benefit without achieving prespecified primary outcomes, including the STICH trials of craniotomy for supratentorial ICH^23,24^ and the MISTIE trial of minimally invasive surgery with thrombolysis.^25^ However, in recent years, studies have suggested that selected patients can derive substantial benefit from ICH evacuation with a significant effect of time, with earlier surgery appearing to be of greater benefit.^26^ Another recent meta-analysis has suggested that minimally invasive haematoma evacuation (rather than full craniotomy) improves functional outcome.^27^ The recent ENRICH trial (NCT02880878; not yet published), compared minimally invasive surgery using the BrainPath approach within 24 h to best medical treatment. The BrainPath device has an atraumatic tip and is used through a trans-sulcal approach to allow access and removal of the haematoma.^28^ ENRICH investigators recruited 300 patients (around two-thirds lobar ICH, one-third anterior basal ganglia) and have reported a significant reduction in death and dependency, only apparent in lobar ICH.^29,30^ Meta-analysis of surgical trials with available end of treatment volume measurements suggest that surgery is of significant benefit only when most of the haematoma is removed, highlighting the critical importance of surgical technique.^31,32^

Ongoing trials (e.g. Dutch ICH Surgery Trial (NCT03608423)) will add to the evidence base for minimally invasive surgery and may allow for robust, evidence-based criteria for haematoma evacuation in ICH. The Decompressive Hemicraniectomy in Intracerebral Hemorrhage (SWITCH, NCT02258919) trial, which recently finished recruitment, will determine whether decompressive hemicraniectomy is beneficial for patients with space-occupying deep ICH. In the meantime, we recommend that each institution develops a consultation process with their local neurosurgical unit based on available experience and resources. ICH severity grading scales provide a standardised means of communicating severity and may be considered as part of local protocols, but caution must be exercised to avoid inappropriate withdrawal of care based on the use of such scales. We provide one example of neurosurgical consultation criteria (Table 1).

#### Insertion of an external ventricular drain (EVD)

Hydrocephalus after ICH is caused by obstruction of the third or fourth ventricles either by blood or by direct compression from the ICH. Inserting an EVD is a common and relatively low-risk procedure, which is considered lifesaving in patients with acute hydrocephalus and is recommended in both the AHA and ESO guidelines.^5,33^

#### Posterior fossa ICH

Cerebellar ICH is associated with increased risks of neurological deterioration due to obstructive hydrocephalus or local mass effect on the brainstem due to its confined anatomical location in the posterior fossa. Data on surgery for posterior fossa ICH, such as suboccipital decompressive craniectomy, haematoma evacuation, or EVD insertion are limited to observational studies.^34^ Surgery may be considered for cases of cerebellar ICH causing brainstem compression and/or acute hydrocephalus.

### Control of glucose

Hyperglycaemia is relatively common in ICH, as in ischaemic stroke, even in patients without diabetes mellitus.^35^ This finding is associated with increased risk of HE, perihaematomal oedema, and worse outcome.^35^ Arguments suggesting that this is a therapeutic target include a post-hoc analysis of the INTERACT2 trial, finding a linear relationship between elevated serum glucose and worse outcome even when adjusting for disease severity.^36^ The best evidence for active treatment comes from cluster-randomised trials, where close glucose management is introduced and compared to standard care. Two trials argue for a benefit: the QASC trial^37^ and the INTERACT3 trial.^3^ In both cases, introducing a bundle of care including hyperglycaemia management significantly improved outcome. It appears that actively monitoring and normalising glucose may reduce brain injury after stroke. However, there was little difference between groups in terms of adjusted mean glucose concentrations over 24 h (−0.5 mmol/L; 95% CI −0.8 to −0.2) in INTERACT3, so the magnitude of contribution of glucose control is uncertain.^3^ Considering the significant resource required to achieve targets and the risk of hypoglycaemia, further evidence is probably needed to support widespread implementation. Pending further studies, we recommend considering the approach used in the INTERACT3 trial:

- Target blood glucose level of 6.1–7.8 mmol/L for nondiabetic patients
- Target blood glucose level of 7.8–10.0 mmol/L for diabetic patients
- Maintain this for 7 days or until hospital discharge.

### Control of temperature

Much as with hyperglycaemia, elevated body temperature (pyrexia) is relatively common in ICH^38^ and is linked to early HE, early neurologic deterioration and poor outcome.^39^ Arguments suggesting that fever is a therapeutic target include a post-hoc analysis of the INTERACT2 trial, finding a linear relationship between pyrexia and worse outcome even when adjusting for disease severity.^36^ As a result, ICH guidelines recommend antipyretic treatment.^5,33^ The best evidence for real-world treatment of pyrexia comes from cluster-randomised trials, where close temperature management is introduced and compared to standard care. Both the QASC trial^37^ and the INTERACT3 trial^3^ argue for a benefit. However, less than 10% of patients in INTERACT3 required any antipyrexia treatment and there was no significant difference in temperature at 1 and 24 h, so the effect size of this intervention is not yet clear. Pending further studies, we recommend considering the antipyrexia approach used in the INTERACT3 trial, which includes temperature checks every 4 h and treatment of any temperature ⩾37.5°C.^3^

## Care bundling

The concept of care bundles was developed by the Institute for Healthcare Improvement in 2001 and is described as ‘a small set of evidence-based interventions for a defined patient segment/population and care setting that, when implemented together, will result in significantly better outcomes than when implemented individually’.^40^ Care bundles may act as a tool to facilitate implementation of evidence-based practice by ensuring that all components of the bundle are considered and delivered effectively to every patient. They have been deployed in other areas of healthcare and are summarised in a recent systematic review and meta-analysis.^41^ The review identified 31 before and after studies suggesting care bundles reduce the risk of negative outcomes but higher level evidence from six randomised trials was less certain.^41^

The concept of a care bundle for ICH was tested at a UK Comprehensive Stroke Centre in 2015–16. The ‘ABC’ care bundle consisted of anticoagulant reversal, BP lowering and a care pathway for neurosurgery consultation. The bundle was associated with a reduction in 30-day case fatality of over 33% (from 35.5% to 24.2%) in a before and after study.^4^ The QASC trial also provided evidence that the ‘fever, sugar, swallow’ (FeSS) care bundle in all stroke patients led to improved outcomes.^37^ Most recently, INTERACT3 has provided higher level evidence for benefit of a care bundle in ICH, incorporating anticoagulant reversal and BP lowering interventions of the ABC bundle and the fever and hyperglycaemia interventions from the FeSS bundle.^3^ Overall, it seems clear that a care bundle approach is beneficial in ICH and should be implemented at all centres caring for ICH patients. Quality improvement methodology is required to ensure optimal implementation.^4^

## Stroke unit care

Determining the optimal environment for ICH patients is critical. Different hospitals may admit such patients to a general medical unit, an intensive care unit, a neurointensive care unit, or a dedicated stroke unit. Stroke unit care is associated with a reduction of the odds of poor outcome,^42^ lower hazard of death, and lower odds of death or dependency and is at least as effective in ICH as for ischaemic stroke.^43,44^ Most recently, a German multicentre retrospective study reported that treatment outside stroke units was associated with higher odds for unfavourable outcome and intrahospital mortality.^45^

The apparent benefit of stroke unit admission is likely related to greater staff expertise, better diagnostic procedures, better nursing care, early mobilisation, prevention of complications, and more effective rehabilitation procedures together with multiparametric telemetry. This means that all ICH patients who do not require intensive care should be preferentially admitted to a stroke unit. Since ICH is a dynamic event with risk of early clinical deterioration occurring over the initial 48 h,^46^ ICH patients should be admitted to a stroke unit as early as possible for close monitoring of physiological parameters and provision of a range of procedures and treatments.^5^

## Conclusions

For many years, randomised trials of single interventions in ICH have failed to meet their primary goal of statistically significant improvement in neurologic outcome. This has led to nihilism in the approach to ICH care.^2^ However, evidence from numerous sources, including randomised trials of bundled care, consistently argue that delivering multiple simultaneous interventions improves functional outcome, with more widespread improvement in supportive care arising from this active approach.^4^ We advocate for the widespread adoption of early bundled care for ICH patients including the optimisation of time-based metrics for BP control and anticoagulation reversal.
